# Temporal Dynamics and Impact of Climate Factors on the Incidence of Zoonotic Cutaneous Leishmaniasis in Central Tunisia

**DOI:** 10.1371/journal.pntd.0001633

**Published:** 2012-05-01

**Authors:** Amine Toumi, Sadok Chlif, Jihene Bettaieb, Nissaf Ben Alaya, Aicha Boukthir, Zaher E. Ahmadi, Afif Ben Salah

**Affiliations:** 1 Laboratory of Medical Epidemiology, Pasteur Institute of Tunis, Tunis, Tunisia; 2 Regional Directorate of Public Health, Sidi Bouzid, Tunisia; Ege University, Turkey

## Abstract

**Background:**

Old world Zoonotic Cutaneous Leishmaniasis (ZCL) is a vector-borne human disease caused by *Leishmania major*, a unicellular eukaryotic parasite transmitted by pool blood-feeding sand flies mainly to wild rodents, such as *Psammomys obesus*. The human beings who share the rodent and sand fly habitats can be subverted as both sand fly blood resource. ZCL is endemic in the Middle East, Central Asia, Subsaharan and North Africa. Like other vector-borne diseases, the incidence of ZCL displayed by humans varies with environmental and climate factors. However, so far no study has addressed the temporal dynamics or the impact of climate factors on the ZCL risk.

**Principal Findings:**

Seasonality during the same epidemiologic year and interval between ZCL epidemics ranging from 4 to 7 years were demonstrated. Models showed that ZCL incidence is raising i) by 1.8% (95% confidence intervals CI:0.0–3.6%) when there is 1 mm increase in the rainfall lagged by 12 to 14 months ii) by 5.0% (95% CI: 0.8–9.4%) when there is a 1% increase in humidity from July to September in the same epidemiologic year.

**Conclusion/Significance:**

Higher rainfall is expected to result in increased density of chenopods, a halophytic plant that constitutes the exclusive food of *Psammomys obesus*. Consequently, following a high density of *Psammomys obesus*, the pool of *Leishmania major* transmissible from the rodents to blood-feeding female sand flies could lead to a higher probability of transmission to humans over the next season. These findings provide the evidence that ZCL is highly influenced by climate factors that could affect both *Psammomys obesus* and the sand fly population densities.

## Author Summary

Old world cutaneous leishmaniasis is a vector-borne disease occurring in rural areas of developing countries. The main reservoirs are the rodents *Psammomys obesus* and *Meriones shawi*. Zoonotic *Leishmania* transmission cycle is maintained in the burrows of rodents where the sand fly *Phlebotomus papatasi* finds the ideal environment and source of blood meals. In the present study we showed seasonality of the incidence of disease during the same cycle with an inter-epidemic period ranging from 4 to 7 years. We evaluated the impact of climate variables (rainfall, humidity and temperature) on the incidence of zoonotic cutaneous leishmaniais in central Tunisia. We confirmed that the risk of disease is mainly influenced by the humidity related to the months of July to September during the same season and mean rainfall lagged by 12 to 14 months.

## Introduction

Zoonotic Cutaneous Leishmaniasis (ZCL) is responsible of considerable morbidity and disfigurement in the Middle East, Central Asia, Subsaharan and North Africa [1], particularly in rural areas. In Tunisia, the epidemic emerged since1982 in Kairouan and expanded to governorates of the center and the south (15/24 governorates were considered as endemic in 2006). So far, more than 100,000 cases were reported mainly from Kairouan, Gafsa and Sidi Bouzid (population size = 1,265,424 from the national census of 2004) [2]. ZCL is considered as one of the most important compulsory diseases in this region. Most of cases are concentrated in rural area where public health human resources and infrastructure are limited. The etiological agent is an Old world *Leishmania* species, *Leishmania major (L. major)*, which is transmitted by the sand fly vector, *Phlebotomus papatasi*. This vector species is highly endophilic (female adults rest indoors) and their biting activity occurs in the evening [3]. Rodents are the reservoir for cutaneous leishmaniasis and include *Psammomys obesus*, *Meriones shawi* and *Meriones libycus*. *Psammomys obesus*, is found in fields of chenopods, a plant of shoals that is its exclusive food source. Transmission is greatest in the summer months (May to September) and infected humans who develop disease (ZCL lesions) tend to do so between October and May [4]. A previous study conducted in Tunisia [5] indicated the importance of environmental changes caused by the development of agriculture and irrigation projects as risk factors for the emergence of ZCL. Climate variability may influence changes in the vector geographical distribution as well as density of rodents' reservoirs is also highly sensitive to availability of food in the environment.

The present study attempted to demonstrate the seasonality of the disease during the same epidemiologic year, to estimate the inter-epidemic interval. The effect of climate factors (rainfall, temperature and humidity) on ZCL incidence was quantified. The findings might help the public health policy makers to reduce the burden of leishmaniasis by more appropriate control programs.

## Materials and Methods

### Study area

**Figure 1:**
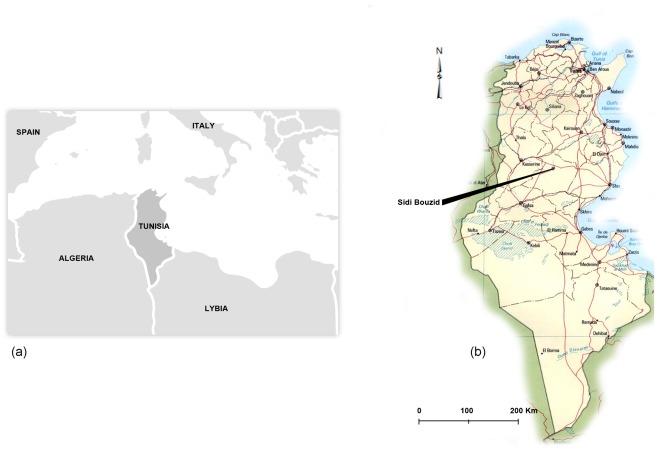
Location of study area. (a) Tunisia Location Within Mediterranean Basin. (b) Location of Sidi Bouzid Governorate Within Tunisia.

This study was conducted in the governorate of Sidi Bouzid (SBZ) (35°02′00″N, 9°30′00″W) located in central Tunisia (Figure 1) where zoonotic cutaneous leishmaniasis emerged as an epidemic since 1991. SBZ is located in central Tunisia in a semi-arid area of 6994 km^2^ and has an estimated population of 412,500 in 2011.

### Data collection

Monthly records of the number of ZCL cases in the whole governorate of SBZ, from January 1991 to December 2007, were collected from the National Control Program of Leishmaniasis (NCPL) of the Regional Directorate of Health of SBZ. The NCPL surveillance strategy relies on passive case detection in 110 health centers as well as active case detection in the schools during epidemics. Case definition is based on parasite confirmation by direct smear and culture in emerging foci and on clinical and epidemiological criteria in old endemic foci. Standardized data collection forms are passed to the NCPL at the regional level on a monthly basis from centers and schools. Data were checked by the data management team at Pasteur Institute of Tunis in collaboration with regional teams, in order to reduce the under reporting bias and the double counts. Queries were sent back to field teams to reconcile inconsistencies and to provide validated forms by consulting the source information.

Meteorological data (monthly average temperature (in °C), average monthly relative humidity (in %) and the cumulative monthly rainfall (in mm)) for the governorate of Sidi Bouzid between 1991 and 2007, were provided by the National Institute of Meteorology (NIM). This data is merged from the meteorological stations of the governorate of Sidi Bouzid by the NIM.

### Statistical methods

#### Seasonality

The seasonality of ZCL incidence was assessed by using a box diagram. The presence of significant seasonality was checked by the combined test of presence of an identifiable seasonal variation described in the following formula:
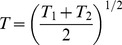
(1)Where T_1_ is the ratio 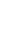, T_2_ the ratio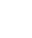 , *F_M_* and *F_S_* are Fisher statistics tests for moving and stable seasonality. If T<1, the null hypothesis is not rejected and we conclude that identifiable seasonality is present as claimed by Lothian and Morry [6]–[7].

#### Seasonal cycles of ZCL

The autocorrelation function (ACF) was used to evaluate the serial correlation of a time series at lags 1, 2, etc and to provide a pattern of seasonality [8].

#### Interannual cycles of ZCL

Interannual cycles of ZCL were measured by the automatic routine seasonal adjustment X-12-ARIMA developed by the US Census Bureau (2011) which is the most appropriate for the structure of the study data (monthly time series for a period over 3 years) [9]. Assuming that the incidence of disease follows an additive model (equation 2), the algorithm X-12-ARIMA had mainly 3 steps. First, the trend-cycle (T_t_) was estimated by applying a moving average 2*12 on ZCL_t_. Second, the seasonal factor was estimated using a moving average of 3*5 on (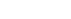). Finally, we estimate the adjusted incidence of disease on seasonal factor by (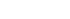) [10].
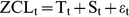
(2)


#### Patterns of association between climate variables and ZCL

The impact of climate variables defined by rainfall, humidity and temperature adjusted for trend and seasonality was assessed using generalized additive model (GAM) [11] and generalized estimating equations (GEE) model [12].

GAM was performed to model the natural logarithm of the expected monthly ZCL incidence as a function of the predictor variables in order to estimate the type of relationship between monthly ZCL incidence and each climate variables. It takes into account the effects of seasonality and trend using penalized regression splines as smoothing functions with 2 degrees of freedom (df) for the climate variables. This technique was implemented by Wood and August in 2002 in R Development Core Team (2009) [13]–[14]. Several GAM candidate models using different time lags (1 lag = 1 month) were generated and tested for the best fit of data in each window. A window corresponds to the effect of rainfall taking into account the effect of the other consecutive lagged rainfalls.

Based on the AIC of GAM models adjusted on seasonality and trend as well as climate variables, the best lag (lag = month) for rainfall was tested up to 14 months. However, for temperature and humidity the lag was fixed to 2 months and their effect was considered only between June and September of the same transmission season. Therefore, ZCL incidence in September and October is associated to these variables in July and August respectively. For the remaining period of disease emergence (December to April), we assigned the value of September.

Best model in each window and between all windows was fitted based on the AIC. A turning point (*TP*) for each non linear relationship between the ZCL incidence and the climate variable was estimated using a maximum likelihood program implemented by Muggeo in R [15].

GEE was used to test the effect of climate variables on ZCL incidence controlling for seasonality and trend. GEE models assumed that observations between years (clusters) are independent while observations within years may be correlated [16]. This correlation structure was assumed to be an auto-regressive of order 1 (AR1). For both GAM and GEE models, the parameter θ of binomial negative distribution of ZCL incidence was estimated by maximum likelihood. This analysis was carried out using the STATA software version 11 (Stata Corporation, College Station, Texas 77845 USA).

## Results

### Descriptive statistics

During the study period, January 1991 to December 2007, the incidence of ZCL ranged widely from 0 to 1608 cases per year in the study area of Sidi Bouzid. Likewise, rainfall showed significant variation while temperature and humidity appeared to be more stable as shown in table 1.

**Table 1:** Yearly descriptive statistics of ZCL incidence, rainfall humidity and temperature during the study period.

Year	ZCL incidence (no)				Rainfall (mm)				Humidity (%)				Temperature (°C)			
	Med	Mean	Min	Max	Med	Mean	Min	Max	Med	Mean	Min	Max	Med	Mean	Min	Max
1991	88.5	289.75	4	1139	20.15	19.6	0	43.3	63	61.33	48	72	14.6	17.2	9.7	27.9
1992	96.5	289.67	2	1608	33.8	30.74	0.2	60.1	61.5	62.42	52	72	15.7	16.24	8.1	25.2
1993	35.5	93.25	2	446	18.4	17.43	0.2	49.4	62.5	61.25	47	72	16.8	18.35	9.1	28.6
1994	18.5	32.17	2	147	14.8	15.62	0	43.2	60	57.58	45	67	18.5	19.63	11.2	30.3
1995	10.5	15	0	50	13.95	23.03	0	65.4	56.5	57.92	47	73	17.5	18.7	10.3	28.3
1996	6.5	12.42	1	39	22.1	27.65	0	66.2	57.5	58.67	49	67	15.9	18.42	10.6	28.8
1997	7	32	2	142	14.45	26.44	.6	143.5	60	58.67	47	70	20.6	19.87	11.6	29.1
1998	9.5	54.17	0	296	6.7	14.81	0	64.9	59.5	59	44	70	19.05	18.97	9.9	28.9
1999	40.5	227.92	0	1321	18.75	27.48	0.1	145.5	61	61.08	51	72	20.8	20.55	10.6	31.2
2000	78.5	222	0	643	3.5	7.33	0	19.4	62	59.08	46	67	18.7	18.88	8.5	28.8
2001	51	164.25	0	553	7.3	16.77	0	63.6	58	58.67	46	70	20.75	20.17	10.8	29.7
2002	42.5	163.17	1	593	10.05	14.58	1.4	44.4	61.5	59.83	47	69	16.7	17.68	9.4	28.4
2003	53.5	204.92	0	1099	26.1	36.67	4.9	87.9	67	63.08	49	71	16.9	18.45	9.7	30.9
2004	92.5	368.5	0	1591	17.75	19.11	0	53.7	60.5	60.42	49	72	15.65	18.27	11.3	29.5
2005	5.5	71.67	0	279	11.15	11.89	0	30	59.5	59.83	46	69	18.5	18.28	9.6	28.4
2006	26.5	47.08	0	149	12.7	21.99	0	71.4	57.5	58.83	43	74	17.55	18.19	8.3	28.5
2007	8	18.67	0	63	17.1	20.97	0	75.6	61	58.75	44	71	16.4	19.21	10.6	29.1

Abbreviations: Med, Median; Min, Minimum;Max, Maximum.

### Seasonal cycles and interannual seasonality

**Figure 2:**
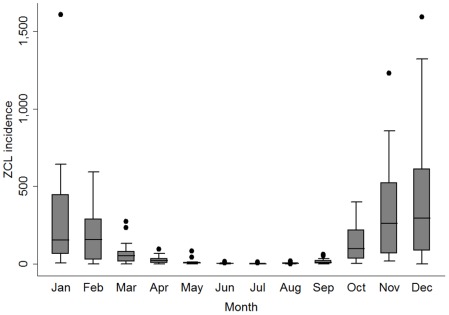
Box plot with monthly ZCL incidence. Data was aggregated from January 1991 to December 2007. The box represents the 25th and 75th percentiles. The median is represented by a solid horizontal line. The whiskers of the graph show the 1st percentile to the 99th percentile. Values lower than the first and greater than the 99th percentile are represented by a point.

**Figure 3:**
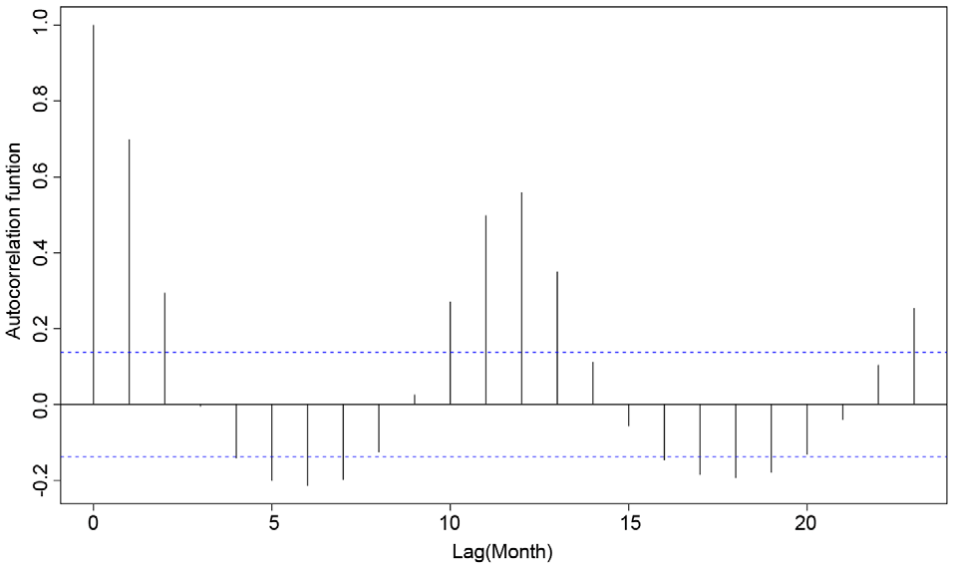
Autocorrelation Function (ACF) of monthly ZCL incidence.

Seasonality of ZCL incidence was significant during the same epidemiologic year defined from the start of vector transmission in May to the end of cases emergence in April the following administrative year (Figure 2). Indeed, the seasonality test rejected the equality of monthly ZCL incidence mean (Fisher's (F_s_) = 19.732, df = 11, p<0.001 and Kruskal-Wallis = 132.9, df = 11, p<0.001). This result demonstrated that the incidence of the disease is significantly higher during the group of months from October to March. The yearly incidence pattern was significantly autocorrelated as shown by the sample autocorrelation function (Figure 3).

**Figure 4:**
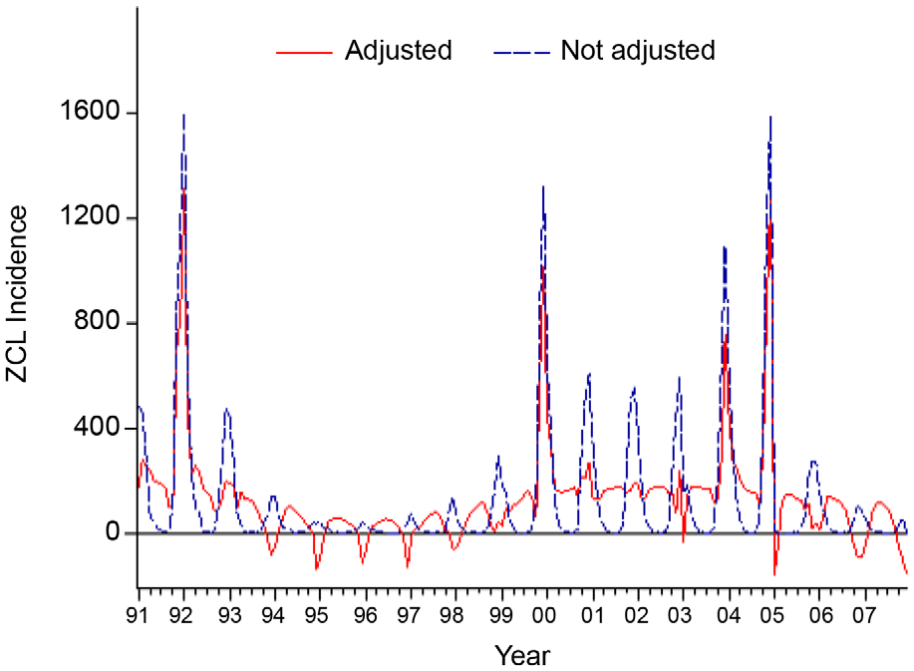
Monthly ZCL incidence adjusted vs not adjusted for monthly seasonality (1991–2007).

The trend of ZCL incidence showed significant peaks in January 1992, December 1999, December 2003 and December 2004 as proven by the moving seasonality test (F_M_ = 5.459, p<0.001). This result confirmed the significant variation of ZCL incidence between years (T = 0.8) with no evidence of residual seasonality in the entire series (F = 1.59, p<0.001). Therefore, the X-12-ARIMA algorithm was run on the original data for the estimation of a monthly adjusted ZCL incidence distribution. Figure 4 shows the trend of the incidence (adjusted and not adjusted for seasonality) of the disease during the whole study period (1991–2007) revealing an inter-epidemic period ranging from 4 to 7 years.

### Climate-ZCL incidence relation

The probability distribution of the ZCL incidence, required for both GAM and GEE models, was over dispersed (Mean = 135.70; Standard deviation, 270.16). A negative binomial distribution fitted adequately the data and permitted to estimate the parameter θ by the maximum likelihood technique (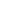 = 0.33, 95% confidence interval (CI): 0.28, 0.38).

**Figure 5:**
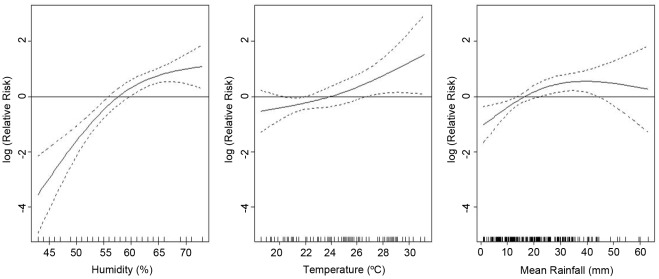
Adjusted relationship between ZCL incidence and humidity, temperature and rainfall. There were no turning points in the relation between temperature/humidity and ZCL incidence. Therefore, we assume these relationships as linear. For rainfall lagged by 12 to 14 months, a turning point equal to 37.34 exists. The range, from each plot, that the climate variables have a positive effect on ZCL incidence is over the zero axis.

The adjusted relationship between the ZCL incidence and climate variables revealed different patterns. For temperature and humidity (lagged by 2 months), a linear relationship provided the best fit while it was rather parabolic for the rainfall (lagged by 12 to 14 months) with a turning point (TP) at 37.34 mm (Figure 5).

Therefore, in order to quantify and test the effect of these climate variables on ZCL incidence, rainfall was divided in two segments and incorporated into the GEE model using two linear terms as following:
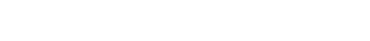


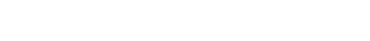
Hence the GEE model is described as follows:
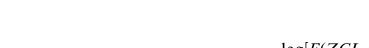
where *ZCL_cases* denotes the numbers of ZCL cases on month *i* (i from 1 to 12) and year *j* (j from 1 to 17). It is the same for Rainfall before the turning point (*R1*), after the turning point (*R2*), humidity (*H*) and temperature (*T*). Confounders were trend and seasonality variables

The results of the GEE model confirmed the significant effect of mean rainfall lagged by 12 to 14 months before the *TP* (p = 0.02) which remained non significant above the *TP* (p = 0.19). Likewise, humidity lagged by 2 months (p = 0.01) exerted a significant effect on ZCL risk in humans. On the other hand, no significant relationship with temperature was detected. The table below provides the percentage of change of ZCL incidence accounted for by unit increase for each climate variable. It shows that the increase by 1 unit in the mean rainfall lagged by 12 to 14 months below the TP contributes to 2.0% increase in the incidence of disease (p = 0.02). However above the turning point, the relationship although not significant (p = 0.19), seems to be negatively associated with high rainfall as the curve decreases after the TP. For humidity above 57.8% and lagged by 2 months the positive effect is more important because 1 unit increase induced 5% increase of the disease incidence (p = 0.01). Temperature effect was not statistically significant (p = 0.17). Table 2 summarizes the percentage of change in disease incidence attributed to climate variables included in the GEE model.

**Table 2:** Adjusted impact of climate variables on the ZCL incidence estimated by GEE model.

Variables	Unit	% Change	95% CI*	P Value
R1_mean14-12_ a	1 mm below 37.34 mm	2.0	0.2, 3.8	0.02
R2_mean14-12_ a	1 mm above 37.34 mm	−3.1	−7.4, 1.7	0.19
Humidity_lag2_ b	1%	5.0	1.2, 10.3	0.01
Temperature_lag2_ b	1°C	8.2	−5.3, 36.9	0.17

*: CI, confidence interval.

a R1 refers to the mean rainfall below 37.34 mm; R2 is the rainfall above the 37.34 mm.

b The lag 2 in humidity and temperature refers to the effect of the months July and August of these climate variables on the ZCL cases that emerged in September and October respectively; ZCL incidence during the remaining months was influenced by the temperature and humidity of the month of September.

## Discussion

As the distribution and behavior of vectors and reservoirs are influenced by environmental conditions, climate variability and change became important determinants of the incidence of many vector-borne diseases such as malaria [17]–[19], dengue [20]–[22], and leishmaniasis [23]–[28]. In South America, climate variability based on El Niño Southern revealed significant effect on leishmaniasis [29]–[30] while in North America, studies have more focused on the influence of temperature and precipitation on the risk and cycles of leishmaniasis [27]–[28]. A significant relationship was found between Mediterranean visceral leishmaniasis and climatic factors [31]–[32]. Old world cutaneous leishmaniasis caused by *L. major* is an increasing problem in Maghreb countries and Eastern Mediterranean region [33]–[37]. Its spatial spread was linked to environmental changes, land use and water development projects such as development of dams and wells for agriculture projects [33]. However, this association has never been, to our knowledge, evaluated quantitatively.

The present study demonstrated significant seasonality within the same year with a highest peak in December for *L. major* cutaneous leishmaniasis. The interval between epidemics derived from the time series of adjusted ZCL data has been shown to range from 4 to 7 years. This result would be partially explained by temporal heterogeneity of the force of infection as proposed by Anderson et al. [38] or other factors related to human populations such as seasonal migration and acquired immunity. The longest period between 1992 and 1999 could be explained by the control intervention based on the reduction of the population of reservoir around the city of Sidi Bouzid by mechanical ploughing in order to reduce the transmission to humans [39]. These results confirm the pertinence of environmental changes as a control option for ZCL. It applies in circumstances where high dense human communities such as urban cities or military camps are surrounded by colonies of *Psammomys obesus*. Unfortunately, this strategy is not feasible in rural area where scattered dwellings are surrounded by heavily infected rodents. Shorter inter-epidemic periods in the following years could be explained by the spread of transmission to surrounding rural area in the governorate of Sidi Bouzid by *Meriones* movements. Indeed, previous work revealed that in addition to *Psammomys obesus*, *Meriones shawi* and *Meriones libycus* are important reservoirs of *L. major* leishmaniasis in Tunisia [40]–[42]. In fact, *Psammomys obesus*, is restricted to fields of chenopods, a plant of shoals that is its primary food source. *Phlebotomus papatasi*, the vector of cutaneous leishmaniasis finds in the burrows of rodents ideal environment and blood meals to maintain the zoonotic *Leishmania* transmission cycle. Previous experience showed that human activities that interfere with the ecologic niche of reservoirs such as *Psammomys* can change the epidemiology of ZCL. Emergence of ZCL epidemics can take place when humans invade the territory of *Psammomys* [5] or the incidence can be reduced when burrows of rodents and chenopods are properly destroyed.

Based on the biologic and epidemiologic reasons [43], time lags for temperature and humidity were fixed with appropriate data transformation in order to have a valid epidemiologic interpretation of statistical results. Besides, it is not recommended to vary the time lags for temperature, humidity and rainfall as many candidate models will be generated and increase the risk to reject wrongly the null hypothesis which corresponds to the type II error. Previous work showed that both temperature and humidity are proven significant environmental conditions for the density and dynamics of *Phlebotomus papatasi* during the transmission period (summer and early autumn) [44]–[45] which is immediately followed by the season of the disease emergence in humans (early autumn to winter).

In the present work, the significant effect of humidity during the transmission season and rainfall of previous year, on incidence of disease was demonstrated and quantified using GAM and GEE. Based on these techniques, we showed for the first time that humidity is more important as a predictor of ZCL incidence than rainfall. Higher rainfall levels would increase the density of chenopods, a halophytic plant, as well as other plants that constitute the food of rodents' reservoirs. Consequently, the reservoir density increases and affects transmission the next season. In fact, rodents benefit from vegetation growth when rainfall is plentiful, but are severely reduced by flooding [46] which might explain the negative association between rainfall above the 37.34 mm and ZCL incidence. On the other hand, the humidity in the summer and autumn enhances transmission by increasing vector density during the same season [47]–[48], a key element in the force of infection through vector capacity, which is analogous to the contact rate in directly-transmitted diseases. Temperature and ZCL incidence were not associated in our model, because most of its effect has been accounted for humidity and rainfall.

Despite the new insight provided by this work, some limitations have to be pointed out. The under reporting bias is hard to rule out completely when dealing with surveillance data. However, the surveillance system of leishmaniasis in the area remained the same during the study period. The high awareness among the community, health decision and policy makers were key elements for sustainability of surveillance and control measures in Sidi Bouzid. It is more likely that the trend of incidence used in time series analysis reproduces a valid evolution of the risk through time.

Prediction of epidemics and early warning remain a high research priority to improve the response of control programs of cutaneous leishmaniasis and the risk assessment for future land use, in the absence of a safe and efficacious vaccine. The present study established, by appropriate modeling techniques, the relationship between old world *L. major* cutaneous leishmaniasis and climate factors.

The findings support the importance of environmental surveillance to detect expansion of rodents' populations following raining seasons and the monitoring of humidity and vector densities to predict epidemics of cutaneous leishmaniasis.
